# Progenitor expansion in *apc *mutants is mediated by Jak/Stat signaling

**DOI:** 10.1186/1471-213X-11-73

**Published:** 2011-12-02

**Authors:** Junji Lin, Xu Wang, Richard I Dorsky

**Affiliations:** 1 Department of Neurobiology and Anatomy, University of Utah School of Medicine, Salt Lake City, UT 84132, USA

**Keywords:** Wnt, APC, Stat3, progenitor, zebrafish

## Abstract

**Background:**

Mutations in *APC*, a negative regulator of the Wnt/ß-catenin pathway, can cause cancer as well as profound developmental defects. In both cases, affected cells adopt a proliferative progenitor state and fail to differentiate. While the upregulation of some target genes of Wnt/ß-catenin signaling has been shown to mediate these phenotypes in individual tissues, it is unclear whether a common mechanism underlies the defects in *APC *mutants.

**Results:**

Here we show that *stat3*, a known oncogene and a target of ß-catenin in multiple tissues, is upregulated in *apc *mutant zebrafish embryos. We further demonstrate that Jak/Stat signaling is necessary for the increased level of proliferation and neural progenitor gene expression observed in *apc *mutants.

**Conclusions:**

Together, our data suggest that the regulation of Jak/Stat signaling may represent a conserved mechanism explaining the expansion of undifferentiated cells downstream of *APC *mutations.

## Background

### Apc loss causes progenitor expansion in development and disease

The Wnt/ß-catenin signaling pathway acts to maintain the undifferentiated progenitor state in multiple epithelial tissues, and overactivation of this pathway is a major contributor to cancer. The tumor suppressor APC normally functions to inhibit Wnt/ß-catenin signaling, and *APC *mutations are oncogenic in tissues such as the colorectal epithelium [1]. During normal embryonic development, Wnt and APC activities are balanced to allow both progenitor cell expansion and differentiation of postmitotic derivatives. Zebrafish embryos homozygous for *apc *mutations exhibit mispatterning and failure of differentiation in multiple tissues including the central nervous system (CNS) [2,3]. Furthermore, in the CNS of other vertebrates, loss of APC function specifically leads to arrest in the neural progenitor state [4]. Despite a clear picture of the cellular phenotypes following loss of APC, the molecular pathways underlying CNS progenitor cell expansion are largely unknown. These pathways may represent good candidates for mediators of oncogenesis in other epithelial cells.

### Transcriptional targets of Wnt signaling mediate *APC *mutant phenotypes

The main downstream output of Wnt/ß-catenin signaling is the transcriptional regulation of target genes, mediated by Lef/Tcf family members. Typically, these targets are repressed by Lef/Tcf factors in the absence of Wnt signaling, and following Wnt activation ß-catenin translocates to the nucleus where it binds to Lef/Tcf proteins and acts as a co-activator. The identification of Wnt/ß-catenin transcriptional targets has thus been a major focus of investigation in past studies of the pathway's role in development and disease. Some identified target genes have been shown to be common targets in both normal embryos and the oncogenic state. For example, *mitf *is a direct target of Lef1 during melanocyte specification, and also plays an important role in melanoma progression downstream of Wnt pathway hyperactivation [5,6]. Similarly, Wnt targets such as *ascl2 *and *lgr5 *may function in both intestinal epithelium homeostasis as well as colon cancer [7,8].

### Stat3 functions synergistically with Wnt signaling in cancer

Like Wnt signaling, the Jak/Stat pathway has been shown to mediate proliferation and tumor growth in cancer. In particular, constitutive Stat3 activity is associated with malignancy in colon cancer [9], the primary carcinoma caused by *APC *mutations. A previous study showed that Wnt signaling can stimulate Stat3 activity during early zebrafish development [10], but the mechanism underlying this activation was not characterized. One potential mechanism of regulation has been suggested by a study in esophageal carcinoma, where *Stat3 *was shown to be a transcriptional target of ß-catenin via Tcf4 [11]. Intriguingly, *Stat3 *has also been suggested to be a target of Wnt signaling in ES cells [12], suggesting that this pathway may represent a developmentally important mechanism. However, the regulatory relationship between Wnt signaling and *Stat3 *activation has not been explored *in vivo *in untransformed tissue.

Here we demonstrate that *stat3 *is a direct transcriptional target of Wnt/ß-catenin signaling in developing zebrafish embryos. We show that increased *stat3 *expression in *apc *mutants correlates with increased proliferation and failure of neuronal differentiation in the developing hypothalamus. Conditional inhibition of Jak/Stat signaling rescues proliferation defects as well as ectopic expression of progenitor markers, but not the general activation of Wnt targets or the complete process of neurogenesis. Together, these data indicate a specific function for Jak/Stat activation in mediating neural progenitor expansion downstream of APC mutations, and suggest a conserved role for this pathway in development and disease.

## Results and Discussion

### *stat3 *is a direct target of the Wnt pathway via Lef1

**Figure 1:**
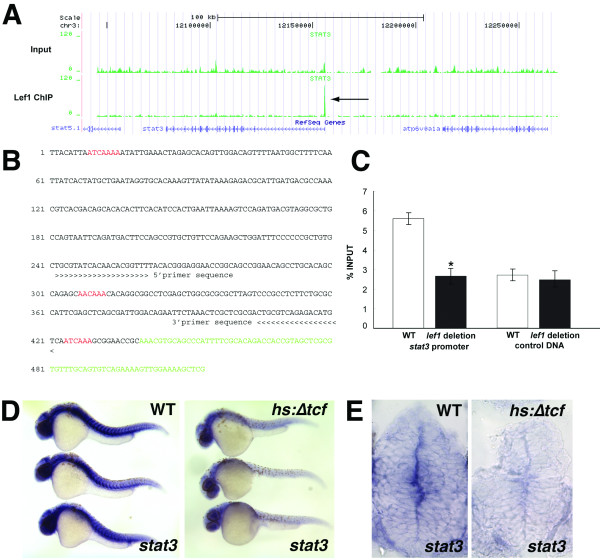
***stat3 *is a direct Lef1 target gene**. **(A) **Density plot from Lef1 ChIP-seq. Sequences from total input chromatin are on top and Lef1 ChIP are below, plotted on the UCSC genome assembly. A peak of Lef1 binding is observed at the *stat3 *promoter (arrow). **(B) **Genomic sequence upstream of *stat3 *transcription start site, identified by Lef1 ChIP-seq. Putative Lef/Tcf binding sites are in red, and mRNA sequence is in green. **(C) **Direct ChIP using Lef1 antibody with primers indicated in panel (B). Mean percent of total input chromatin by qPCR from 3 independent experiments is shown. Precipitation from wild-type 36 hpf chromatin is significantly enriched over chromatin from *lef1 *deletion mutant embryos. ChIP from a control genomic region without putative binding sites shows no enrichment. Error bars = s.d., *p < 0.05 by student's t-test. **(D,E) ***stat3 *expression is decreased 8 hours after ubiquitous expression of the constitutive repressor ΔTcf. Cross-sections through the hypothalamus are shown in **(E)**. Both wild-type embryos (left) and siblings expressing *hs:Δtcf *(right) were heat-shocked at 28 hpf and processed for *stat3 *in situ hybridization at 36 hpf.

We have previously shown that Wnt signaling, mediated by the transcriptional effector Lef1, is required for hypothalamic neurogenesis in the zebrafish brain [13]. To identify transcriptional targets of the Wnt pathway, we performed ChIP-seq analysis using a Lef1 antibody. Immunoprecipitation was performed using chromatin from whole 36 hours post-fertilization (hpf) embryos, corresponding with a time of high *lef1 *expression in the hypothalamus. After deep sequencing of precipitated chromatin, we observed high enrichment of the *stat3 *promoter region compared to total input as well as chromatin from *lef1 *deletion mutant embryos. The genomic sequence identified by ChIP-seq (Figure 1A) contains several putative Lef/Tcf consensus binding sites (Figure 1B), and we confirmed the direct interaction with Lef1 using ChIP followed by quantitative PCR (Figure 1C).

We next tested whether the endogenous expression of *stat3 *in the zebrafish embryo depends on Wnt-mediated transcription. We used a transgenic inducible repressor of Lef/Tcf target genes (*hs:ΔTcf*) to globally inhibit pathway activity in vivo. 28 hpf embryos were heat shocked for one hour, allowed to recover until 36 hpf, and then processed for in situ hybridization. We observed a qualitative decrease in *stat3 *expression throughout embryos expressing ΔTcf, including in the hypothalamus (Figure 1D,E). Together, these results suggest that *stat3 *is a direct transcriptional target of the Wnt pathway.

### *stat3 *expression and Stat3 phosphorylation are increased in *apc *mutants

**Figure 2:**
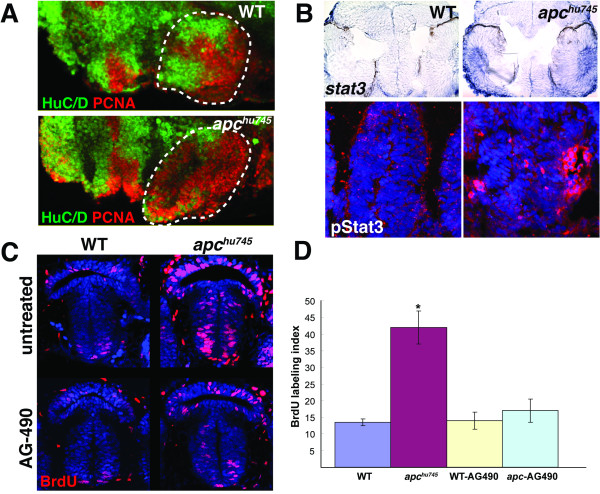
**Jak/Stat signaling mediates proliferation in the *apc *mutant hypothalamus**. **(A) **Proliferating cells are increased and neurogenesis is decreased in the *apc *mutant hypothalamus. Lateral confocal projections are shown with the neuronal marker HuC/D in green and the proliferation marker PCNA in red. Anterior is to the left and the hypothalamus is indicated by the dotted lines. **(B) **Both *stat3 *mRNA expression and pStat3 levels are increased in the *apc *mutant hypothalamus at 36 hpf. In situ hybridization for *stat3 *mRNA (top) and immunohistochemistry for pStat3 (bottom) are shown in transverse cryosections. **(C) **BrdU incorporation in the hypothalamus is increased in *apc *mutants, and restored to wild-type levels by AG-490 treatment. Embryos were labeled with BrdU for 1 hour and fixed for analysis at 36 hpf. Confocal projections from the ventral brain surface through the 36 hpf hypothalamus are shown. BrdU immunohistochemistry is red, TO-PRO-3 nuclear stain is in blue. **(D) **The BrdU labeling index is significantly increased in the *apc *mutant hypothalamus compared to wild-type siblings, and restored to wild-type levels by AG-490 treatment. Error bars = s.d., p < 0.05 by student's t-test.

Previous studies have reported multiple developmental defects in the CNS of *apc *mutant zebrafish embryos, including axon pathfinding errors [14], loss of normal brain patterning [3], and expansion of the putative retinal stem cell zone [2]. An additional striking phenotype that we observed in mutant embryos was a dramatic increase in proliferating cells particularly in the hypothalamus, accompanied by a dramatic decrease in differentiated neurons (Figure 2A). An earlier study identified *stat3 *as a marker that was increased in *apc *mutant embryos in the putative retinal stem cell zone and the hypothalamus [2]. We examined *stat3 *expression throughout the *apc *mutant embryo and observed a qualitative increase in mRNA levels, with specific enrichment in known CNS progenitor zones including the hypothalamus (Figure 2B). Quantitative PCR analysis of *apc *mutant embryos showed an increase in the level of *stat3 *mRNA of 5.34 ± .09 fold (s.d., n = 3, p < 0.05 by student's t-test) compared to wild-type siblings. We also found a qualitative increase in pStat3 immunostaining in the *apc *mutant hypothalamus compared to control embryos (Figure 2B), suggesting that *stat3 *mRNA levels may normally limit the signaling output of this pathway. Based on the known roles of Stat3 function in progenitor cell maintenance, these results raised the possibility that increased Jak/Stat signaling might underlie some of the progenitor differentiation defects present in the *apc *mutant brain.

### Increased proliferation in *apc *mutants can be rescued by blocking Jak/Stat signaling

In other tissues, *APC *mutations and Stat3 hyperactivation can both lead to increased cell proliferation. To quantify the proliferative increase in *apc *mutant zebrafish, we performed short-pulse (1 hour) BrdU labeling in wild-type and mutant embryos. At 36 hpf, significantly more cells within the developing hypothalamus of *apc *mutant embryos incorporated BrdU than in wild-type siblings (Figure 2C,D). These data are consistent with an increased number of progenitor cells in the CNS of *apc *mutants compared to wild-type embryos.

We next tested whether inhibition of Jak/Stat activity could reverse the increased proliferation found in *apc *mutants. To block Jak/Stat signaling, we used the Jak2 inhibitor AG-490, which has been demonstrated to prevent Stat3 phosphorylation in many other experimental systems including zebrafish [15] and allowed us to bypass early developmental defects resulting from *stat3 *knockdown. When wild-type embryos were incubated in 40µm AG-490 from 24-36 hpf, we did not observe a significant change in the BrdU labeling index compared to untreated controls (Figure 2C,D). In contrast, AG-490 incubation completely reversed the increase in proliferation observed in *apc *mutant embryos, restoring the BrdU labeling index to wild-type levels (Figure 2C,D). Together, these data indicate that Jak/Stat signaling is required for increased proliferation in *apc *mutant brains. Our observations of increased *stat3 *mRNA expression in *apc *mutants suggest that Stat3 levels may be limiting in the developing brain, and that regulation by the Wnt pathway may control the ability of Jak/Stat signaling to drive cell proliferation.

### Increased progenitor marker expression in *apc *mutants requires Jak/Stat activity

**Figure 3:**
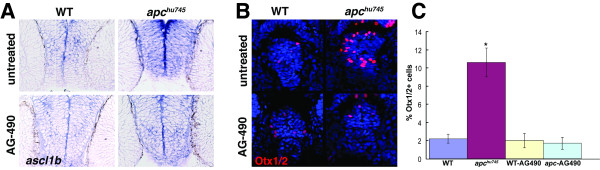
**Jak/Stat signaling mediates progenitor marker expression in the *apc *mutant hypothalamus**. **(A) **Expression of *ascl1b*, a neural progenitor marker, is qualitatively increased in the *apc *mutant hypothalamus, and restored to wild-type levels by AG-490 treatment. Transverse cryosections at 36 hpf are shown. **(B) **Expression of Otx1/2, a hypothalamic progenitor marker, is increased in the *apc *mutant hypothalamus, and restored to wild-type levels by AG-490 treatment. Confocal projections from the ventral brain surface through the 36 hpf hypothalamus are shown. Otx1/2 immunohistochemistry is red, TO-PRO-3 nuclear stain is in blue. **(C) **The percent of Otx1/2+ cells is significantly increased in the *apc *mutant hypothalamus compared to wild-type siblings, and restored to wild-type levels by AG-490 treatment. Error bars = s.d., p < 0.05 by student's t-test.

Because proliferation is closely linked to the progenitor cell phenotype in the developing CNS, we wanted to determine whether other markers of neural progenitors were also increased in *apc *mutants and whether this increase depends on Jak/Stat activity. We first examined the expression of *ascl1b*, which encodes a proneural bHLH transcription factor essential for neurogenesis. Using in situ hybridization, we found that *ascl1b *mRNA levels were qualitatively increased in the *apc *mutant hypothalamus at 36 hpf (Figure 3A). Incubation in 40µM AG-490 from 24-36 hpf was able to eliminate this increase and restore *ascl1b *expression to wild-type levels in *apc *mutants (Figure 3A), suggesting that increased proneural gene expression is mediated by Jak/Stat activity.

In the zebrafish retina, *otx1 *expression marks the putative stem cell zone of the ciliary margin, and is expanded in *apc *mutants [2]. Otx1 and Otx2 are also expressed in the developing vertebrate hypothalamus and label neural progenitors in the zebrafish hypothalamus. We observed increased *otx1 *mRNA expression in the hypothalamus of *apc *mutants (not shown), and to provide a more quantitative measurement, we examined the number of cells labeled with an antibody that recognizes both Otx1 and Otx2. Within the hypothalamus, *apc *mutants showed a significant increase in Otx1/2-positive cells at 36 hpf (Figure 3B,C), and this increase was rescued to wild-type levels by AG-490 incubation (Figure 3B,C). These data suggest that cells may be arrested in an Otx-positive progenitor state following *apc *inactivation, and that Jak/Stat function mediates this arrest.

### Inhibition of Jak/Stat activity is not sufficient to rescue neurogenesis in *apc *mutants

**Figure 4:**
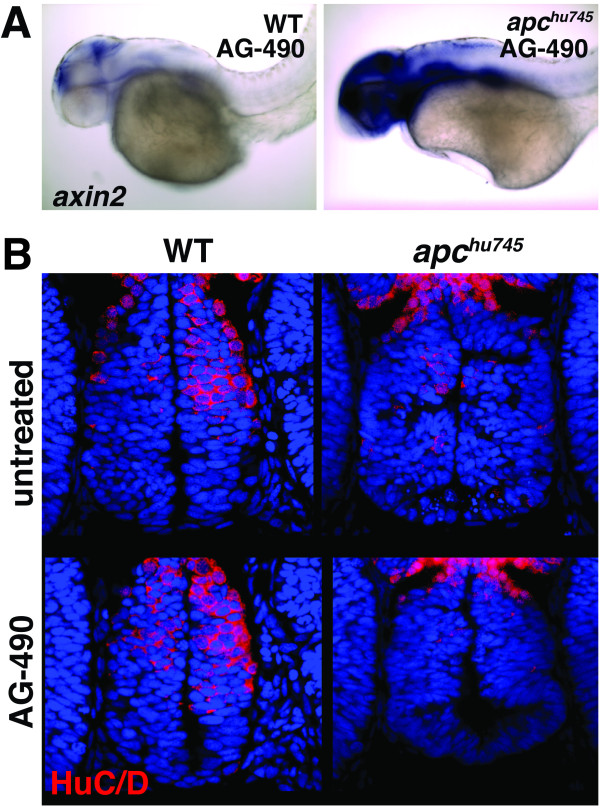
**Jak/Stat signaling does generally mediate Wnt-responsive gene expression or the entire neurogenesis program in *apc *mutants**. **(A) ***axin2 *mRNA expression, a general marker for Wnt/ß-catenin target gene activation, is increased in *apc *mutants treated with AG-490 at 36 hpf, compared to controls. Lateral whole-mount views at 36 hpf are shown. **(B) **Expression of HuC/D, a marker of differentiated neurons, is decreased in the *apc *mutant hypothalamus, and remains decreased after AG-490 treatment. Confocal projections from the ventral brain surface through the 36 hpf hypothalamus are shown. HuC/D immunohistochemistry is red, TO-PRO-3 nuclear stain is in blue.

While Jak/Stat activity is required for the expansion of CNS progenitor characteristics downstream of *apc *inactivation and *stat3 *transcription, we hypothesized that this pathway is not likely to mediate all outputs of Wnt activation. Indeed, when we examined the expression of the Wnt target gene *axin2*, we observed a strong increase in mRNA expression that was not rescued by AG-490 incubation (Figure 4A). This result indicates that many transcriptional targets of Wnt/ß-catenin signaling are likely to be independent of Jak/Stat activity, and that these targets may act in parallel pathways. Furthermore, while AG-490 incubation could rescue increases in proliferation and progenitor gene expression, it was insufficient to restore neurogenesis in *apc *mutants. The loss of HuC/D expression observed in the hypothalamus was still seen in embryos after incubation in AG-490 (Figure 4B), suggesting that neural progenitors were still unable to differentiate into neurons. Therefore, other Stat3-independent targets of APC must be important for regulating the full program of differentiation. These could possibly include Wnt-independent APC targets, as has been demonstrated previously in other studies [16].

## Conclusions

Here we have shown that *stat3 *is a direct transcriptional target of Wnt signaling in the developing embryo, and that Jak/Stat signaling mediates the expansion and maintenance of CNS progenitor characteristics downstream of Wnt hyperactivation in *apc *mutants. Together, our data suggest that transcriptional regulation of *stat3 *may represent a general mechanism linking Wnt pathway overactivation to the expansion of undifferentiated cells in the disease state.

At higher doses of AG-490, we were able to completely eliminate both proliferation and progenitor marker expression in wild-type embryos (not shown). Combined with the endogenous expression pattern of *stat3*, and the fact that ΔTcf can repress *stat3 *in wild-type embryos, this suggests that a Wnt/Stat3 pathway may also play an important role in normal CNS development.

## Methods

### Zebrafish maintenance and embryo culture

Embryos were obtained from natural spawning of wild-type (AB*), *Tg(hsp70l:tcf3-GFP)^w26^*, *Df(LG01:lef1,msxb)^x8^*, and *apc^hu745 ^*mutant zebrafish and were staged according to Kimmel et al., [17]. *lef1 *deletion and *apc *mutant embryos were identified by morphology and *hs:Δtcf *embryos were identified by expression of a GFP fusion protein. All embryos were raised at 28.5°C and fixed in 4% PFA for analysis. 28 hpf *hs:Δtcf *embryos were heat shocked for 1 hour at 37°C, then allowed to recover at 28.5°C until 36 hpf. To block Jak/Stat signaling, embryos were treated with 40 uM AG-490 (Enzo) beginning at 24 hpf. For BrdU labeling, 35 hpf embryos were incubated in 10 mM BrdU in 15% DMSO for 30 minutes on ice, washed and allowed to recover for 1 hour at 28.5°C before fixation.

### ChIP and qPCR

ChIP analysis was performed as described previously [18] with the following modifications. One hundred embryos at 36 hpf were dechorionated and fixed in 1% PFA in PBS for 15 minutes at room temperature, and then lysed in cell lysis buffer [10 mM Tris (pH 8.1), 10 mM NaCl, 0.5% NP- 40, and protease inhibitors] and nuclear lysis buffer [50 mM Tris-Cl (pH 8.1), 10 mM EDTA, 1% SDS and proteinase inhibitors] by pipetting. For each immunoprecipitation, 5 ug of anti-Lef1 antibody [13] was conjugated to 30 ul Dynabeads (Invitrogen) prior to applying nuclear extract. A detailed protocol is posted at: https://wiki.zfin.org/display/prot/ZFIN+Protocol+Wiki. Precipitated DNA fragments were purified and submitted for Illumina sequencing at the University of Utah HSC Core Facility and sequences were mapped to zebrafish genome (assembly zv7).

For qPCR analysis of ChIP fragments, total input chromatin and Lef1 immunoprecipitated chromatin from wild-type and *Df(LG01:lef1,msxb)^x8 ^*mutant siblings was used. For qPCR analysis of *stat3 *mRNA levels, total RNA was isolated from 42 hpf wild-type and *apc^hu745 ^*mutants using an RNAeasy extraction kit (Qiagen) followed by DNase treatment. cDNA was synthesized by SuperScript II reverse transcriptase (Invitrogen), and *stat3 *levels were normalized to *beta actin *cDNA. Quantitative real-time PCR was performed at the University of Utah HSC Core Facility.

Primers used for *stat3 *ChIP qPCR are: **5'-TGCGTATCACAACACGGTTT-3' 5'-ACATGTCTCTGACGCAGTCG-3' **Primers used for *stat3 *cDNA qPCR are: **5'-CCGACTGGAAGAGGAGACAG-3' 5'-GCTGGACGGTGCTGAATAAT-3'**

### In situ hybridization

Whole mount in situ hybridization was performed as described previously [13]. Probes for *stat3 *[19] and *otx1 *[20] were obtained from T. Piotrowski. Probes for *ascl1b *and *axin2 *were synthesized in our laboratory. Following staining, whole embryos were mounted in 80% glycerol and imaged on a dissecting microscope, or embedded in plastic, sectioned, and imaged on a compound microscope.

### Immunohistochemistry

For BrdU and PCNA detection, fixed embryos were incubated for 1 hour in 2N HCl. Immunostaining was performed as described previously [21]. Antibodies were obtained from the following sources: anti-BrdU (AbD Serotec, 1:500), anti-HuC/D (Molecular Probes, 1:500), anti-OTX1/2 (Chemicon, 1:500), anti-PCNA (Sigma, 1:1000), anti-pStat3 (Tyr708, MBL, 1:1000), and secondary antibodies conjugated to Alexa Fluor 647 (Invitrogen). Following immunohistochemistry, embryos were counterstained with TO-PRO-3 (Invitrogen), and whole brains were dissected for imaging. Embryos were mounted in Fluoromount-G (Southern Biotech), and confocal images were acquired using an Olympus FV1000 microscope.

## Authors' contributions

J.L. conducted all experiments except the PCNA analysis of *apc *mutants, qPCR for *stat3*, and pStat3 staining, which were performed by X.W. R.I.D. provided oversight for the entire study and wrote the manuscript. All authors read and approved the final manuscript.
